# Supplementary material for: mRNA-LNP COVID-19 Vaccine Lipids Induce Complement Activation and Production of Proinflammatory Cytokines: Mechanisms, Effects of Complement Inhibitors, and Relevance to Adverse Reactions
Source: Int J Mol Sci. 2024 Mar 22;25(7):3595. doi: 10.3390/ijms25073595 (PMC11012056; doi:10.3390/ijms25073595)
Supplement: Supplementary file 1 [file ijms-25-03595-s001.zip › ijms-2888276-supplementary.pdf]

Table S1. Lipid composition of Comirnaty and Doxebo

	Comirnaty*			Doxebo**			
	Lipid	mg/mL	%	Lipid	mg/mL	%	Dox/CMT
Ionizable lipid (ALC-0315)	((4-hydroxybutyl) azanediyl) bis (hexane-6,1-diyl)bis(2-hexyldecanoate))	1.43	55.8	none	0	0	
Phospholipid	1,2-distearoyl-sn-glycero-3-phosphocholine	0.3	11.7	Fully hydrogenated soy phosphatidylcholine	9.58	60	5.1
Cholesterol	Cholest-5-en-3β-ol	0.67	26	Cholest-5-en-3β-ol	3.19	20	0.8
PEGylated lipid (ALC-0159)	(2-((polyethylene glycol)-2000)-*N,N*-ditetradecylacetamide))	0.17	6.5	N-(carbonyl-methoxypolyethylene glycol 2000)-1,2-distearoyl-sn-glycero-3-phosphoethanolamine	3.19	20	3.1
Total		2.57	100		15.96	100	**6.2**
Membrane forming phospholipids			18			80	**4.4**

*Data from Ref. 110. **, Data based on the lipid composition of Doxil[108]

Table S2. SARS-CoV-2 mRNA does not activate human complement

	C4d (μg/ml)	Bb (μg/ml)	sC5b-9 (μg/ml)
PBS	2.6	10	2.6
Comirnaty	5.1	17.2	5.5
SARS-CoV mRNA*	2.7	9.6	1.7
Zymosan	8.6	47.1	142.2

Experiments conducted in a serum different from those applied in Figure 4. All experimental details are the same as described for Figure 4. *, 20 μg/mL

Table S3. CD40L (moCD40L gene) mRNA sequence, 1,200 nt

AGGCCGGCGGGTTTCTGACATCCGGCGGGTTTCTGACATCCGGCGGGTTTCTGACATCCGGCGGGTTTCTGACAT<br>CCGGCGGGTTTCTGACATCCGGCGGGTTTCTGACATCCGGCGGGTTTCTGACATCCGGCGGGTTTCTGACATCCG<br>GCGGGTTTCTGACATCCGGCGGGTTTCTGACATTCACAACCAGGCCTCCACAACCATGGTCGAGACATACAGCCA<br>GCCCAGCCCCAGAAGTGTGGCCACAGGCCTGCCCGCCAGCATGAAAATCTTTATGTATCTGCTGACCGTGTTCCT<br>GATCACCCAGATGATCGGCAGCGTGCTGTTCGCCGTGTATCTGCACAGACGGCTGGACAAGGTGGAGGAAGAAG<br>TGAATCTGCACGAGGACTTCGTGTTCATCAAGAAGCTGAAGCGCTGCAACAAGGGCGAGGGCAGCCTGAGCCT<br>GCTGAACTGCGAGGAAATGAGAAGGCAGTTCGAGGACCTGGTGAAGGACATCACCCTGAACAAAGAGGAAAA<br>GAAAGAAAACAGCTTCGAGATGCAGAGAGGCGACGAAGATCCTCAGATCGCAGCCCACGTGGTGTCTGAGGCC<br>AACAGCAACGCCGCCAGCGTGCTGCAGTGGGCCAAGAAAGGCTACTACACCATGAAGTCCAACCTGGTGATGC<br>TGGAAAACGGCAAGCAGCTGACCGTGAAGAGAGAAGGCCTGTACTACGTGTACACCCAGGTGACATTCTGCAG<br>CAACAGAGAGCCCAGCAGCCAGAGGCCTTTCATCGTGGGCCTGTGGCTGAAGCCTAGCAGCGGCAGCGAGAGA<br>ATCCTGCTGAAGGCCGCCAACACCCACAGCAGCAGCCAGCTGTGCGAGCAGCAGTCTGTGCACCTGGGCGGCG<br>TGTTCGAACTGCAGGCAGGCGCCAGCGTGTTCGTGAACGTGACCGAGGCCAGCCAGGTGATCCACAGAGTGGG<br>CTTCAGCAGCTTCGGCCTCCTGAAGCTGTAACTCGAGTGTTTTGGCTGGGTTTTTCCTTGTTCGCACCGGACACCT<br>CCAGTGACCAGACGGCAAGGTTTTTATCCCAGTGTATATTGTCGACAAAAAAAAAAAAAAAAAAAAAAAAAA<br>AAAAAAAAAAAAAAAAAAAAAAAAAAAAAAAAAAAAAAAAAAAAAAAAAAAAAAAAAAAAAAAAAAAA<br>AAAAAAAAAAAAAAAAAAAAAAAAAAAAA

*batch: RG-mRNA-moCD40L-200806)-N1UCC-Si

Table S4. Luciferase (rstsFluc gene) mRNA sequence (1,450 nt)

AGGCCGGCGGGTTTCTGACATCCGGCGGGTTTCTGACATCCGGCGGGTTTCTGACATCCGGCGGGTTTCTGACAT<br>CCGGCGGGTTTCTGACATCCGGCGGGTTTCTGACATCCGGCGGGTTTCTGACATCCGGCGGGTTTCTGACATCCG<br>GCGGGTTTCTGACATCCGGCGGGTTTCTGACATTCACAACCAGGCCTCCACAACCATGGAAGACGCCAAAAACA<br>TAAAGAAAGGCCCGGCGCCATTCTATCCTCTAGAGGATGGAACCGCTGGAGAGCAACTGCATAAGGCTATGAAG<br>AGATACGCCCTGGTTCCTGGAACAATTGCTTTTACAGATGCACATATCGAGGTGAACATCACGTACGCGGAATAC<br>TTCGAAATGTCCGTTCGGTTGGCAGAAGCTATGAAACGATATGGGCTGAATACAAATCACAGAATCGTCGTATGC<br>AGTGAAAACTCTCTTCAATTCTTTATGCCGGTGTTGGGCGCGTTATTTATCGGAGTTGCAGTTGCGCCCGCGAACG<br>ACATTTATAATGAACGTGAATTGCTCAACAGTATGAACATTTCGCAGCCTACCGTAGTGTTTGTTTCCAAAAAGGG<br>GTTGCAAAAAATTTTGAACGTGCAAAAAAAATTACCAATAATCCAGAAAATTATTATCATGGATTCTAAAACGGA<br>TTACCAGGGATTTCAGTCGATGTACACGTTCGTCACATCTCATCTACCTCCCGGTTTTAATGAATACGATTTTGTAC<br>CAGAGTCCTTTGATCGTGACAAAACAATTGCACTGATAATGAATTCCTCTGGATCTACTGGGTTACCTAAGGGTGT<br>GGCCCTTCCGCATAGAGCTCTCTGCGTCAGATTCTCGCACGCCAGAGATCCAATTTTTGGCAATCAAATCGCTCCG<br>GATACTGCGATTTAAGTGTTGTTCCATTCCATCACGGTTTTGGAATGTTTACTACACTCGGATATTTGATATGTGGA<br>TTTCGAGTCGTCTTAATGTATAGATTTGAAGAAGAGCTGTTTTTACGATCCCTTCAGGATTACAAAATTCAAACTGC<br>GTTACTAGTACCAACCCTATTTTCATTCTTGGCCAAAAGTACTCTGATTGACAAATACGATTTATCTAATTTACACG<br>AAATTGCTTCTGGGGGCGCACCTCTTTCGAAAGAAGTCGGGGAAGCGGTTGCAAAACGCTTCCATCTTCCAGGG<br>ATACGACAAGGATATGGGCTCACTGAGACTACTAGTGCTATTCTGATTACACCCAAGGGGGATGATAAACCGGGC<br>GCGGTCGGTAAAGTTGTTCCATTTTTTGAAGCGAAGGTTGTGGATCTGGATACCGGGAAAACGCTGGGCGTTAAT<br>CAGAGAGGCGAATTATGTGTCAGAGGACCTATGATTATGTCCGGTTATGTAAACAATCCGGAAGCGACCAACGCC<br>TTGATTGACAAGGATGGATGGCTACATTCTGGAGACATAGCTTACTGGGACGAAGACGAACACTTCTTCATAGTT<br>GACCGCTTGAAGTCTTTAATTAAATACAAAGGATATCAGGTGGCCCCCGCTGAATTGGAATCGATATTGTTACAAC<br>ACCCCAACATCTTCGACGCGGGCGTGGCTGGTCTTCCCGACGATGACGCCGGTGAACTTCCCGCCGCCGTTGTTG<br>TTTTGGAGCACGGAAAGACGATGACTGAAAAAGAGATCGTGGATTACGTCGCCAGTCAAGTAACAACCGCGAA<br>AAAGTTGCGCGGAGGAGTTGTGTTTGTGGACGAAGTACCGAAAGGTCTTACCGGAAAACTCGACGCAAGAAAA<br>ATCAGAGAGATCCTCATAAAGGCCAAGAAGGGCGGAAAGTCCAAATTGTAACTCGAGTGTTTTGGCTGGGTTTTT<br>CCTTGTTCGCACCGGACACCTCCAGTGACCAGACGGCAAGGTTTTTATCCCAGTGTATATTGTCGACAAAAAAAA<br>AAAAAAAAAAAAAAAAAAAAAAAAAAAAAAAAAAAAAAAAAAAAAAAAAAAAAAAAAAAAAAAAAAAAAAAAAAAAAAAA<br>AAAAAAAAAAAAAAAAAAAAAAAAAAAAAAAAAAAAAAAAAAAAAAAAAAAA

*batch: RG-mRNA-pETRCC-tsrsfluc_200623-N1UCC_Si).

Table S5. Cre-recombinase (Cre) gene mRNA sequence (2,086 nt)*

AGGCCGGCGGGTTTCTGACATCCGGCGGGTTTCTGACATCCGGCGGGTTTCTGACATCCGGCGGGTTTCTGACAT<br>CCGGCGGGTTTCTGACATCCGGCGGGTTTCTGACATCCGGCGGGTTTCTGACATCCGGCGGGTTTCTGACATCCG<br>GCGGGTTTCTGACATCCGGCGGGTTTCTGACATTCACAACCAGGCCTCCACAACCATGTCTAACTTGTTGACTGTT<br>CATCAGAATCTTCCTGCCTTGCCCGTAGATGCTACAAGTGACGAAGTACGGAAGAATCTCATGGATATGTTTCGA<br>GATAGACAAGCTTTTTCAGAGCATACATGGAAGATGCTGTTGTCTGTGTGCCGAAGCTGGGCTGCTTGGTGTAAA<br>TTGAATAACAGGAAATGGTTTCCTGCAGAACCTGAAGATGTACGGGACTATCTGCTGTATCTGCAAGCTAGAGGA<br>CTTGCCGTCAAGACAATCCAGCAACATCTCGGACAACTGAATATGTTGCATAGAAGATCCGGCCTGCCTCGGCCA<br>AGTGACTCAAACGCTGTGTCACTGGTGATGCGAAGGATACGGAAAGAAAATGTAGACGCCGGGGAAAGGGCTA<br>AACAAGCTTTGGCTTTCGAACGAACTGACTTTGATCAGGTTAGGTCCTTGATGGAGAACTCTGACCGATGTCAAG<br>ACATCAGAAATCTCGCCTTCCTGGGAATTGCCTATAACACATTGCTGAGAATTGCTGAAATAGCAAGGATAAGAG<br>TTAAAGACATCAGCAGGACTGATGGCGGCAGGATGCTCATTCACATTGGGCGCACTAAGACTCTCGTCTCTACTG<br>CTGGCGTCGAAAAAGCTTTGTCTCTCGGTGTCACTAAACTCGTCGAGCGCTGGATCTCTGTTTCTGGTGTCGCTGA<br>CGACCCCAACAACTACTTGTTTTGCCGCGTGAGGAAGAACGGGGTTGCCGCCCCCAGTGCTACATCTCAACTGTC<br>AACCCGAGCACTCGAAGGAATCTTCGAGGCTACACACCGATTGATATACGGGGCTAAGGATGATAGTGGGCAGC<br>GGTATCTGGCTTGGAGTGGGCATTCTGCTCGAGTTGGTGCAGCACGAGACATGGCCCGAGCCGGCGTGTCAATAC<br>CCGAAATAATGCAGGCTGGAGGATGGACTAACGTGAATATTGTTATGAATTATATTCGCAACCTGGATAGTGAGAC<br>TGGTGCAATGGTACGGTTGCTCGAAGATGGTGACTAACTCGAGTGTTTTGGCTGGGTTTTTCCTTGTTCGCACCGG<br>ACACCTCCAGTGACCAGACGGCAAGGTTTTTATCCCAGTGTATATTGTCGACAAAAAAAAAAAAAAAAAAAAA<br>AAAAAAAAAAAAAAAAAAAAAAAAAAAAAAAAAAAAAAAAAAAAAAAAAAAAAAAAAAAAAAAAAAA<br>AAAAAAAAAAAAAAAAAAAAAAAAAAAAAAAAAAA

*batch: RG-mRNA-pETRCC-Cre-200723_N1UCC_Si)
